# Determination of Triglycidyl Isocyanurate in Workplace Air

**DOI:** 10.3390/ijerph16224455

**Published:** 2019-11-13

**Authors:** Anna Jeżewska, Joanna Kowalska

**Affiliations:** Central Institute for Labour Protection – National Research Institute, Czerniakowska 16, 00-701 Warsaw, Poland; jokow@ciop.pl

**Keywords:** TGIC, tris (2,3-epoxypropyl) isocyanurate, mutagenic substance, air pollution, occupational exposure, chromatography, health sciences, environmental engineering

## Abstract

Triglycidyl isocyanurate (TGIC) is a white solid in powder or granular form. TGIC does not occur naturally in the environment. It is intentionally manufactured and used as a crosslinking agent or hardener to produce polyester powder coatings. TGIC may cause genetic defects. This article presents the method of TGIC determination in workplace air using high-performance liquid chromatography (HPLC) with a diode-array detector (DAD). The method is based on the collection of TGIC present in the air on a polypropylene filter, extraction with acetonitrile, and chromatographic analysis of the solution obtained in this way. The determination was carried out in the reverse-phase system (mobile phase: acetonitrile: water) using an Ultra C18 column. The measurement range is 2 to 40 µg/m^3^ for a 720 liters air sample. Limit of detection (LOD) is 23 ng/m^3^ and limit of quantification (LOQ): 70 ng/m^3^. The method can be used for assessing occupational exposure to TGIC and associated risk to workers’ health.

## 1. Introduction

Triglycidyl isocyanurate (TGIC) (CAS No: 2451-62-9) is a solid with a specific gravity of 1.2–1.7 g/cm^3^. It is produced from cyanuric acid and epichlorohydrin in an alkaline solution of 1,4-dioxane. It is mainly used for hardening polyester powder paints, which, thanks to TGIC, are characterized by very good functional properties, such as mechanical and chemical resistance, resistance to UV radiation, and maintaining gloss [1,2]. 

TGIC is highly irritating to the eyes. It can be harmful to the central nervous system, kidneys, liver, lungs, and digestive tract. Repeated or prolonged contact may cause an allergic skin reaction or asthma. It may cause a heritable genetic defect in human reproductive cells [3,4,5,6,7,8,9,10,11,12]. TGIC is a mutagenic substance of category 1B (Muta. 1B). Table 1 shows the classification defining the type of hazard caused by this substance, according to the European Commission Regulation [13].

Exposure to TGIC can occur during its production and use, especially during coating applications when TGIC-containing powder coatings are sprayed on metal by hand or during cleaning the equipment or paint booths [2].

From the resources of the Central Register of Data on Exposure to Carcinogenic or Mutagenic Substances, Mixtures, Agents, or Technological Processes, maintained by the Nofer Institute of Occupational Medicine, Łódź, Poland, it transpires that in Poland in 2016, only 11 employees were occupationally exposed to TGIC. These data are probably underestimated. Maximum permissible concentration (MAC) for this substance in the workplace air has not yet been established in Poland. MAC values applicable in selected countries are presented in Table 2.

TGIC determination in the air of the working environment was carried out with different methods. In the method developed by the Occupational Safety and Health Administration (OSHA), air containing TGIC is filtered through glass fiber filters coated with hydrobromic acid. The absorbed substance is desorbed with dimethylformamide (DMF), derivatized with heptafluorobutyric acid, and the obtained solution is then determined by gas chromatography with an electron capture detector (GC-ECD). The TGIC limit of detection is 23.3 µg/m^3^ [15]. 

The Health and Safety Executive (HSE) method describes the measurement of TGIC concentrations in air and in powder coatings. In this method, air samples are collected on a silanized glass fiber filter, extracted with tetrahydrofuran or acetonitrile with the addition of a mobile phase, and the solution is determined using high-performance liquid chromatography (HPLC) with spectrophotometric detection (UV/VIS). The measurement range of this method is from 5 to 400 µg/m^3^. The detection limit for TGIC is 0.9 μg/m^3^ [16].

In another method, the ultraperformance liquid chromatography coupled with a tandem mass spectrometer (UPLC-MS/MS) is used for the determination of air samples containing TGIC. Air samples are collected on an Accu-cap^TM^ system containing a polyvinyl chloride filter from which TGIC is extracted using a mixture of acetone and acetonitrile. After dilution of the sample with water, the solution is analyzed using UPLC-MS/MS. Limit of detection is 0.2 µg/m^3^, and limit of quantification is 0.7 µg/m^3^ [17].

This paper presents the methodology of TGIC determination in workplaces air, which was prepared in accordance with the guidelines included in the European standard EN-482:2012+A1:2015 [18]. Because the MAC value for TGIC was not established in Poland, it was decided that TGIC will be determined in the range of concentrations from 2 to 40 µg/m^3^. 

## 2. Materials and Methods 

### 2.1. Equipment

Throughout the study, an Agilent Technologies (Agilent Technologies, Waldbronn, Germany) series 1200 liquid chromatograph with a diode detector (DAD) and a fluorescence detector (FLD) in an online configuration were used. The samples were dosed using an autosampler. ChemStation software was used for process control, determination, and data acquisition. The following equipment was also used. Ultra C18 column; dimensions: (250 x 4.6 mm) with dp = 5 μm, with pre-column, dimensions: 10 × 4.0 mm (Restek, Bellefonte, PA, USA). GilAir 5 (Sensidyne, Clearwater, FL, USA) aspirator for air sampling. WL-2000 (JWElectronic, Warsaw, Poland) mechanic shaker for TGIC recovery from the filter.

### 2.2. Material and Reagents

The following reagents were used in the study: TGIC from Aldrich (Aldrich Chemistry, Tokyo, Japan), cyanuric acid (Aldrich Chemistry, Shanghai, China), 1,4-dioxane (Merck, Darmstadt, Germany), epichlorohydrin (Sigma–Aldrich, Steinheim, Germany), acetonitrile (J. T. Baker, Deventer, The Netherlands), high-purity water produced by the Milli-Q apparatus (Millipore, Bedford, MA, USA). HPLC grade reagents were used throughout the study.

Materials: filters made of poly(propylene), FIPRO with a diameter of 25 mm (Textile Institute, Lodz, Poland), filters made of poly(vinyl chloride), PVC with a diameter of 25 mm (Sensidyne, St. Petersburg, FL, USA), poly(ethylene terephthalate), Zefluor™, PTFE filters with a diameter of 25 mm (Pall Laboratory, Mexico, Mexico), and glass fiber filters, GF/A with a diameter of 25 mm (Whatman, Maidstone, UK). IOM (Institute of Occupational Medicine) sampler (SKC Inc, 863 Valley View Rd, PA, USA) for air sampling. 

## 3. Methodology

### 3.1. Sample Preparation

Air samples containing TGIC (720 L) were collected on a polypropylene filter (FIPRO) placed in an IOM type sampler. The sampling time was 6 hours. After the air sampling, the filter was placed in the Erlenmeyer flask. One point five milliliters of acetonitrile was used for the recovery of TGIC deposited on the filter. The flasks were shaken for 30 minutes. The solution from above the filter was chromatographed using an Ultra C18 HPLC (Restek, Bellefonte, PA, USA) column with pre-column. 

### 3.2. Chromatographic Conditions

**Figure 1:**
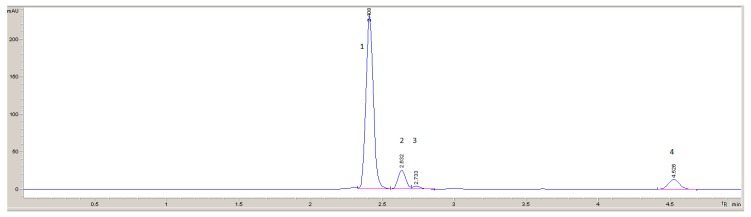
Chromatogram of a standard solution of triglycidyl isocyanurate (TGIC) and coexisting substances. 1. TGIC, 2. epichlorohydrin, 3. 1,4-dioxane, 4. cyanuric acid. high-performance liquid chromatography–diode-array detector (HPLC-DAD), analytical wavelength (λ) = 205 nm.

The temperature of the measurements was 23 °C. Acetonitrile:water (98:2, v/v) was used as the mobile phase. The flow rate of the mobile phase was 1 mL/min. The volume of the injected sample was 50 μl. A DAD detector with an analytical wavelength of λ = 205 nm was used for detection. Such conditions allowed the TGIC to be determined in the presence of other substances that may also be in the workplace air, such as epichlorohydrin, 1,4-dioxane, or cyanuric acid (Figure 1).

### 3.3. Recovery Rate Studies

The study of the TGIC recovery rate from the polypropylene filter (FIPRO) was conducted in the following way: The filters (6 pieces each) were coated with 1.44 µg TGIC (dosing 50 µL TGIC solution in acetonitrile at 28.8 µg/mL), 14.4 µg TGIC (dosing 50 µL TGIC solution in acetonitrile at 288 µg/mL), and 28.8 µg TGIC (dosing 50 µL TGIC solution in acetonitrile at 576 µg/mL). The filters were dried, 720 L of air was passed through them, and the recovery was carried out with 1.5 mL of acetonitrile, shaking the contents for 30 min. Solutions from above the filters were determined chromatographically under the conditions previously established. TGIC determination was also carried out in comparative solutions made in the same way but without a filter. The determined average recovery rates for the three measurement series were from 0.99 to 1.03. 

### 3.4. Calibration and Precision

Calibration was made for six TGIC standard solutions in acetonitrile in the concentration range from 0.96 to 19.2 µg/mL, which were analyzed chromatographically. To evaluate the precision of calibration determinations, three series of eight working solutions were prepared with TGIC concentrations successively at 1.92 µg/mL (first series), 9.6 µg/mL (second series), and 19.2 µg/mL (third series). Chromatographic measurements of two of each solution were performed under the same conditions as for calibration determinations. For each of three series of standard solutions, standard deviations and the coefficients of variation were calculated.

### 3.5. Method Validation

The method was validated according to the requirements of EN 482:2012+A1:2015 [18]. The limit of detection and the limit of quantification were based on the results of the analysis of ten independent peak area measurements with the TGIC retention time for three independently prepared blank samples. To calculate the value of the limit of detection (LOD), following dependence was used:

(1) $$LOD=\frac{3,3\cdot s_{o}}{b},$$

where:

*b* – slope coefficient of the calibration curve;
*s_o_* – standard deviation of results obtained for a series of blank samples.

To calculate the LOQ dependency, this relation was used:

*LOQ* = *3·LOD.* (2)

The overall precision of the examination (*V_c_*), allowing for laboratory measuring ranges and techniques employed for sampling, has been calculated using the formula:

(3) $$V_{c}=\sqrt{V_{z}^{2}+V_{p}^{2}},$$

where:

*Vp* – is the precision of the sampling device (*Vp* = ± 5%);
*Vz* – mean precision of three levels of ranges, which has been calculated using the formula:

(4) $$V_{z}=\sqrt{\frac{\sum (n_{j}-1)\cdot V_{i}^{2}}{\sum (n_{j}-1)}},$$

where:

*n_j_* is the number of duplicated samples (*nj* = 8); and
*Vi* is the coefficient of variation for a given level of concentration.

The overall precision of the examination value (Vc) is taken into account in the calculation of relative total uncertainty. The relative expanded uncertainty is obtained by multiplying the relative total uncertainty with a coverage factor of 2.

## 4. Results and Discussion

The studies were conducted to determine the conditions of collected air samples containing TGIC to determine its concentration in the air at workplaces. TGIC is a solid under normal conditions. Therefore, filters should be used to separate it from the air. The following filters were selected for testing: PVC (poly(vinyl chloride)), PTFE (poly(ethylene terephthalate)), FIPRO (poly(propylene)), and GF/A (glass fiber), which are dedicated to air sampling by the individual dosimetry method. This method is used to assess the individual exposure of a worker by measuring the concentration of a harmful substance with a sampler placed in the breathing zone. The principles of taking air samples at the workplace are specified in the PN-Z-04008-7:2002 standard [19]. Air samples should be taken: in the breathing zone, individually for each worker, for the entire period of their stay at the workplace, or for at least 75% of the duration of a shift.

### 4.1. Tests of the Efficiency of TGIC Recovery from Filters

The filter suitable for collecting TGICs from the air was selected after preliminary testing of the recovery rate of TGICs from the filters: PVC, PTFE, FIPRO, and GF/A. Fifty microliters of TGIC solution in acetonitrile (96 µg/mL) was applied to filters (n = 3) and placed in a glass container and dried. The dried filters were transferred to conical flasks and extracted with 1.5 mL of acetonitrile. After shaking the flasks (30 min), the solutions from above the filters were analyzed chromatographically under the conditions described above.

The PVC filters stuck to the glass container in which they were dried, making it impossible to transfer them entirely to conical flasks for acetonitrile extraction. For this reason, they were not suitable for further research. The lowest recovery factor was obtained for PTFE filters. However, the recovery rate values for FIPRO and GF/A filters were similar (Table 3). 

FIPRO filters were selected for further research due to their lower cost than GF/A filters. These filters are non-hygroscopic, and the solutions obtained after extraction from FIPRO filters do not require additional filtration before analysis, which reduces the cost of analysis.

### 4.2. Sampling

The FIPRO filter in the IOM (Institute of Occupational Medicine) sampler was used to collect air samples containing the TGIC because it effectively captures particles with an aerodynamic diameter of up to 100 µm in a way similar to inhaling particles through the nose and mouth in a working environment [20]. To determine the air sampling conditions, the system consisted of a FIPRO filter with the substance applied (28.8 µg) placed in an IOM sampler and a GilAir 5 pump with a constant volume air flow rate controlled by a rotameter. 

The air passed through each filter at the flow rate recommended by the manufacturer of the sampler for the collection of inhalable fraction, i.e., 2 L/min for 6 hours (720 L of air) according to the sampling strategy of PN-Z-04008-7:2002 [19] and EN 689:2018+AC:2019 [21]. Comparative samples were taken from filters through which air was not passed through. The results of the tests are presented in Table 4.

The results obtained indicate that TGIC had stopped on the FIPRO filter.

### 4.3. Recovery Rate Studies

Recovery rates were calculated by comparing the results of the analysis from extracted samples (peak area) at three concentration levels with the results from non-extracted standards (for which the recovery rate is 1). The results of the tests are presented in Table 5. The average recovery rate was 1.01. The relative standard deviation was lower than 10% for all the values obtained.

### 4.4. Calibration and Precision

**Figure 2:**
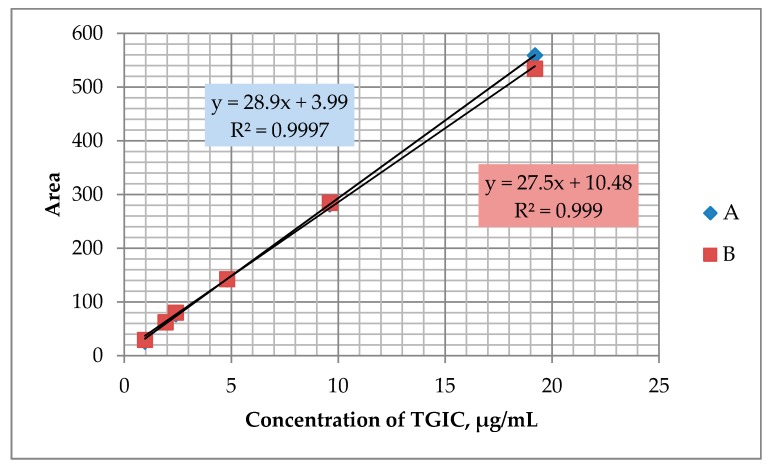
Plot of the relationship between peak area and TGIC concentration in calibration solutions obtained using two methods: A—from standard solutions; B—from filters.

Calibration was made for a series of standard working solutions with concentrations of 0.96 µg/mL, 1.92 µg/mL, 2.4 µg/mL, 4.8 µg/mL, 9.6 µg/mL, and 19.2 µg/mL TGIC in acetonitrile, which were analyzed chromatographically (Figure 2. of curve A). The linearity of the calibration curve is characterized by the value of the correlation coefficient. Correlation coefficient for this curve was *r* = 0.9999. 

The second curve (Figure 2. curve B) was obtained after application to FIPRO filters of 50 µL of TGIC solutions in acetonitrile in concentrations of: 28.8 µg/mL, 57.6 µg/mL, 72 µg/mL, 144 µg/mL, 288 µg/mL, and 576 µg/mL. Solutions obtained after acetonitrile extraction (1.5 mL) were analyzed chromatographically. The correlation coefficient characterizing the linearity of the second standard curve was *r* = 0.9995. With this method of calibration (second calibration curve), there was no need to determine recovery efficiency since the TGIC filters taken from the workplace were subjected to the same procedure of preparation for analysis as the filters with the substance applied in the laboratory for the calibration curve. Possible losses of the substance resulting from the preparation of the sample for analysis will be identical for the sample as for the preparation of the standard curve. In this case, the recovery rate is always 1.

To evaluate the precision of calibration determinations, three series of eight working solutions of concentration were prepared: 1.92 µg/mL, 9.6 µg/mL, and 19.2 µg/mL. Chromatographic measurements of two of each solution were performed under the same conditions as for calibration determinations. The values characterizing the precision of calibration determinations are presented in Table 6. The coefficients of variation for subsequent concentration levels were, respectively: 2.61%; 1.91%; 0.66%. The overall precision of the examination (according to formula 3) was 5.35%.

### 4.5. Sample Storage

The stability of the air samples was tested successively and after one, five, and seven days of storage in a desiccator and refrigerator. The results showed that the samples stored in the desiccator at room temperature (22 °C ± 2 °C) and in the refrigerator (4 °C ± 2 °C) were stable for at least 7 days. The results are shown in Table 7.

### 4.6. Validation

The method was validated according to the requirements contained in the European Standard EN 482:2012+A1:2015 [18]. The obtained validation data are presented in Table 8. The relative expanded uncertainty for this method was acceptable and amounted to 24%.

## 5. Conclusions

As a result of the study, a suitable sorbent (polypropylene filter) for TGIC absorption from the air was selected, which in an IOM sampler allows air samples in the breathing zone of a worker to be taken according to the principles of personal dosimetry, and thus, gives the possibility of obtaining the most reliable results for the assessment of inhalation exposure of workers to this substance. The presented preparation of the calibration curve, including the stage of sample preparation for analysis, eliminates the stage of determining the recovery rate and thus reduces the analysis time and the consumption of reagents. The method is precise, accurate, and it meets the criteria for procedures for measuring chemical agents listed in Standard No. EN 482:2012+A1:2015. The method developed may be used by occupational hygiene laboratories to determine TGIC concentrations in the air in the range from 2 to 40 µg/m^3^ to enable the exposure assessment of this substance.

## Figures and Tables

**Table 1:** Classification identifying the type of risks caused by Triglycidyl isocyanurates (TGICs) [13].

Hazard Class and Category Codes	Hazard Statements Codes
Muta. 1B	H340 May cause genetic defects
Acute Tox. 3	H331 Toxic if inhaled
Acute Tox. 3	H301 Toxic if swallowed
STOT RE 2	H373 May cause damage to organs through prolonged or repeated exposure
Eye Dam. 1	H318 Causes serious eye damage
Skin Sens. 1	H317 May cause an allergic skin reaction
Aquatic Chronic 3	H412 Harmful to aquatic life with long-lasting effects

**Table 2:** TGIC hygienic standard values for eight-hour exposure [14].

Country	Limit Value—Eight Hours [mg/m^3^]
Australia	0.08
Belgium	0.05
Canada—Ontario	0.05
Finland	0.1
Ireland	0.05
New Zealand	0.08
Spain	0.05
United Kingdom	0.1

**Table 3:** Comparison of filters (TGIC recovery from filters).

Type of Filter	Average Area of TGIC Peaks from Recovered Solutions	Average Area of TGIC Peaks from Comparative Solutions	Recovery Rate	Average Recovery Rate
PTFE	29.6	71.75	0.41	0.37
	22.8		0.32	
	26.8		0.37	
FIPRO	70.7	71.75	0.99	0.98
	71.3		0.99	
	69.9		0.97	
GF/A	70.2	71.75	1.10	0.98
	70.5		1.09	
	71.2		1.10	

**Table 4:** Adsorption of TGIC on FIPRO (poly(propylene)) filters.

Mass of TGIC on the Filter [µg]	Air Flow Rate [L/min]	Area of the TGIC Peaks in Solution:	
		After Recovery from the Filter	Comparative
28.8	2	861.9	874.5
		880.1	
		869.2	

**Table 5:** Determination of the TGIC recovery rate from the FIPRO filters.

Concentration of TGIC Solution [µg/mL]	Average Area of Peaks from Recovered Solutions	Average Area of Peaks from Comparative Solutions	Relative Standard Deviation [%]	Average Recovery Rate
0.96	28.7	28.8	4.43	0.99
9.6	273.1	265.1	3.34	1.03
19.2	509.0	505.0	1.35	1.01

**Table 6:** Parameters characterizing the precision of chromatographic determination.

Parameter	Measurement Series		
	I	II	III
Concentration of the solution [μg/mL]	1.92	9.6	19.2
Average value of peak area	60.09	301.74	567.23
Standard deviation	1.57	5.77	3.75
Coefficient of variation [%]	2.61	1.91	0.66

**Table 7:** Results of the stability test of samples containing TGIC.

Filter No.	Storage Place	Storage Time [Number of Days]	Average Peak Area of the TGIC	Two-Filter Average	Standard Deviation
1	desiccator	1	293.30	293.35	0.1
2			293.40		
1	refrigerator	1	279.00	279.85	1.2
2			280.70		
1	desiccator	5	273.45	272.10	1.9
2			270.75		
1	refrigerator	5	282.25	278.10	5.9
2			273.95		
1	desiccator	7	300.60	301.38	1.1
2			302.15		
1	refrigerator	7	302.65	300.53	3.0
2			298.40		

**Table 8:** Validation parameters of the method for the determination of TGIC.

Parameter	Value
Measurement range	0.002–0.04 mg/m^3^
Sampled air volume	720 L
Range of calibration curve	0.96–19.2 µg/mL
Limit of detection (LOD)	11.2 ng/mL (23.3 ng/m^3^)
Limit of quantitation (LOQ)	33.6 ng/mL (70 ng/m^3^)
Overall precision of examination	5.35%
Relative total uncertainty	12%
Relative expanded uncertainty	24%
